# Acute gastric dilatation after excessive consumption of traditional Chinese medicine: a case report

**DOI:** 10.3389/fmed.2025.1584032

**Published:** 2025-07-02

**Authors:** Tao Zeng, Zi-liang Chen, Yao-hui Zhou, Wei-qi liu, Jian Chen, Jian-hui Lu, Jia-hao Lin

**Affiliations:** ^1^Zhongshan Hospital of Traditional Chinese Medicine Affiliated to Guangzhou University of Traditional Chinese Medicine, Zhongshan, Guangdong, China; ^2^Guangzhou University of Chinese Medicine, Guangzhou, Guangdong, China

**Keywords:** gastroenterology, emergency medicine, radiology frequent vomiting, excess of Chinese medicine, acute gastric dilatation

## Abstract

**Introduction:**

Acute gastric dilatation (AGD) is a rare but clinically significant condition characterized by abnormal enlargement of the stomach. It can lead to serious complications such as gastric necrosis, perforation, and respiratory failure if not promptly managed. AGD has been associated with mechanical obstructions, binge eating, and systemic conditions like diabetes mellitus. However, AGD induced by excessive consumption of traditional Chinese medicine (TCM) has not been previously reported.

**Patient concerns:**

A 40-year-old male with a history of chronic dyspepsia presented to the emergency department with recurrent vomiting. Over the 3 days prior to admission, he self-reported consuming approximately 3.5 liters of TCM liquid (about 1.1–1.3 liters per day). This attempt to alleviate his chronic dyspepsia symptoms failed to show the expected therapeutic effect. The patient denied experiencing headache, dizziness, chest pain, palpitations, abdominal pain, or diarrhea.

**Diagnosis:**

Abdominal CT revealed significant gastric distension, and subsequent endoscopy showed pyloric ulcer with stenosis, gastric retention, chronic atrophic gastritis, and a relaxed cardia. Laboratory investigations indicated metabolic alkalosis, electrolyte imbalances, and signs of tissue hypoxia.

**Interventions:**

The patient was immediately managed with nasogastric tube decompression, anti-infection therapy, gastric mucosal protection, fluid resuscitation, parenteral nutritional support, fasting, and gastrointestinal decompression.

**Outcomes:**

The patient’s symptoms and signs notably improved after these interventions. A follow-up CT scan demonstrated improved gastric dilation. At a 4-week follow-up, the patient reported complete resolution of vomiting and resumed normal oral intake. Repeat endoscopy showed healed pyloric ulcers and improved gastric motility. No adverse events (e.g., rehospitalization or medication intolerance) were reported during a 3-month follow-up period.

**Conclusion:**

This case highlights the necessity of including AGD in the differential diagnosis for patients presenting with frequent vomiting after excessive consumption of TCM. It underscores the importance of thorough evaluation to prevent misdiagnosis and severe complications. The case also emphasizes the need for caution when using TCM, especially in patients with organic lesions or pyloric obstruction.

## Introduction

Acute gastric dilatation (AGD) is a rare yet clinically significant condition marked by abnormal enlargement of the stomach. If not promptly identified and managed, AGD can result in serious complications such as gastric necrosis, perforation, and respiratory failure due to the distended stomach, which can occasionally be fatal (1–4). AGD has been associated with multiple etiological factors, including mechanical causes (e.g., gastric cancer, scarring from peptic ulcers, or superior mesenteric artery syndrome) and systemic conditions (e.g., diabetes mellitus). In the realm of non-obstructive factors, binge eating is a relatively well-documented cause (5–7). Additionally, systemic conditions such as diabetes mellitus and systemic sclerosis have been linked to the development of AGD (8).

To the best of our knowledge, while AGD cases associated with binge eating have been reported, there are no previous reports of AGD attributed to excessive consumption of TCM. We present a case of a 40-year-old male patient with a history of chronic dyspepsia. He had been using TCM to regulate gastrointestinal function and aid digestion. However, the TCM failed to show the expected therapeutic effect in this instance. Instead, he continued to consume large amounts of the TCM over a short period, leading to acute gastric dilatation (AGD) and even shock. Gastroscopy revealed pyloric stenosis and a relaxed cardia, which may have contributed to the exacerbation of AGD.

## Case presentation

A 40-year-old male patient was admitted to the emergency department with the chief complaint of recurrent vomiting. The patient reported a 3-day history of recurrent vomiting and belching, with progressive worsening culminating in severe symptoms on the day of admission. The TCM consumption occurred throughout this period, peaking on the final day with an intake of 1.3 liters. He denied experiencing headache, dizziness, chest pain, palpitations, abdominal pain, or diarrhea. Upon arrival, the patient was found to be alert and oriented, with no signs of altered mental status. His past medical history was notable for chronic dyspepsia, for which he had been intermittently taking TCM to regulate gastrointestinal function. However, this time he self-reported consuming approximately 3.5 liters of TCM liquid over 72 h. Additionally, he had a history of gastric ulcer over 10 years ago. He had no history of alcohol abuse. Notably, he had never been previously admitted for AGD.

Upon admission, the patient, a 40-year-old male, was measured at a height of 175 cm and had a body weight of 75 kg. On admission, his vital signs were: temperature 36.6°C, blood pressure 91/53 mmHg (hypotensive), tachycardia (pulse 134 bpm), and tachypnea (respiratory rate 24/min). The physical examination findings were documented with particular attention to abdominal and systemic signs of decompensation. On admission, the patient exhibited tachycardia (134 bpm) and hypotension (91/53 mmHg), consistent with hypovolemic shock secondary to persistent vomiting. Abdominal inspection revealed visible distension without surgical scars or peristaltic waves. Palpation demonstrated generalized tenderness, most pronounced in the epigastrium, though without rebound tenderness or guarding. Auscultation detected hypoactive bowel sounds (1–2/min), correlating with the CT findings of gastric hypomotility. Notably, the absence of abdominal rigidity helped exclude perforation, while decreased skin turgor and dry mucous membranes confirmed moderate dehydration. These findings, combined with the laboratory results (metabolic alkalosis, hypokalemia) and imaging features, formed a cohesive clinical picture of AGD complicated by fluid and electrolyte losses. Cardiopulmonary auscultation revealed clear lung fields and regular heart sounds without murmurs. Neurological examination showed no focal deficits. Skin turgor was decreased, indicating mild dehydration. Laboratory investigations revealed the following results: Blood routine examination showed a hemoglobin level of 11.9 g/dL and a white blood cell (WBC) count of 16.21 × 10^9^/L. The platelet count was 498 × 10^9^/L. Thyroid function tests indicated that the levels of free triiodothyronine (FT3) were 5.15 pmol/L, free thyroxine (FT4) were 24.25 pmol/L, and thyroid-stimulating hormone (TSH) was 7.55 mIU/mL. Blood glucose was measured at 10.45 mmol/L. Blood urea nitrogen (BUN) was 17.10 mmol/L, and serum creatinine was 227 μmol/L. Electrolyte levels revealed a potassium concentration of 2.44 mmol/L, sodium level of 130 mmol/L, and chloride level of 77.8 mmol/L. Liver function tests showed that alanine aminotransferase (ALT) was 7 U/L, aspartate aminotransferase (AST) was 21 U/L, and total bilirubin was 19.18 μmol/L. Additionally, the D-dimer level was 0.5 μg/mL. On the second day following admission, the white blood cell count improved to 5.04 × 10^9^/L. Arterial blood gas analysis revealed a pH of 7.58, which is above the normal range of 7.35–7.45, indicating metabolic alkalosis. The partial pressure of carbon dioxide (PaCO2) was 51 mmHg, exceeding the normal range of 35–45 mmHg, suggesting respiratory compensation. The bicarbonate (HCO3-) level was 46.5 mEq/L, which is above the normal range of 22–27 mEq/L, further supporting the diagnosis of metabolic alkalosis. The lactate level was 7.31 mmol/L, which is above the normal range of 0.50–1.70 mmol/L, suggesting tissue hypoxia or impaired lactate metabolism. The findings are consistent with acute gastric dilatation, which can lead to metabolic alkalosis due to vomiting and electrolyte imbalances.

**FIGURE 1:**
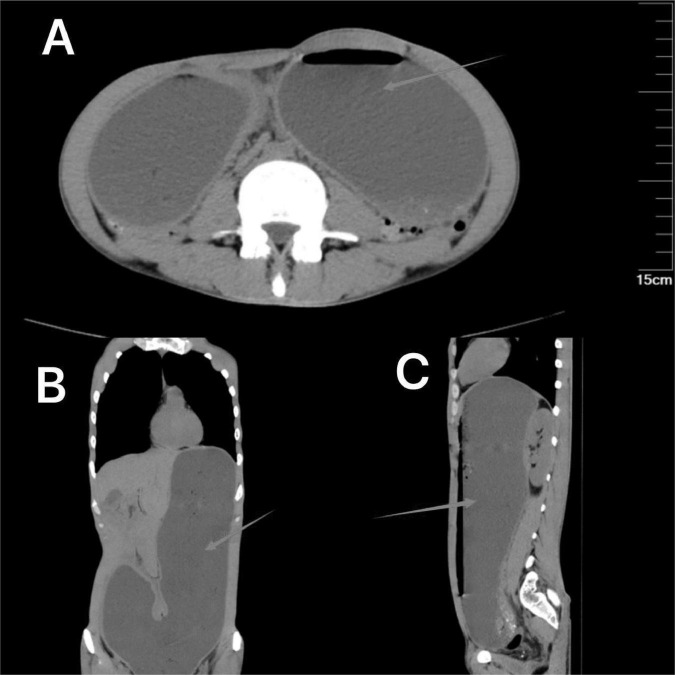
Non-contrast abdominal CT on admission showing gastric distension (arrows). **(A)** (Axial view) shows the expanded gastric silhouette with fluid levels. **(B)** (Coronal view) and **(C)** (sagittal view) further illustrate the distended stomach, confirming the presence of fluid.

The non-contrast CT scan of the abdomen revealed marked gastric dilation with fluid accumulation, suggesting a possible obstruction at the pyloric region without evidence of distal duodenal or further gastrointestinal tract involvement (Figure 1). Pyloric obstruction was highly suspected.

**FIGURE 2:**
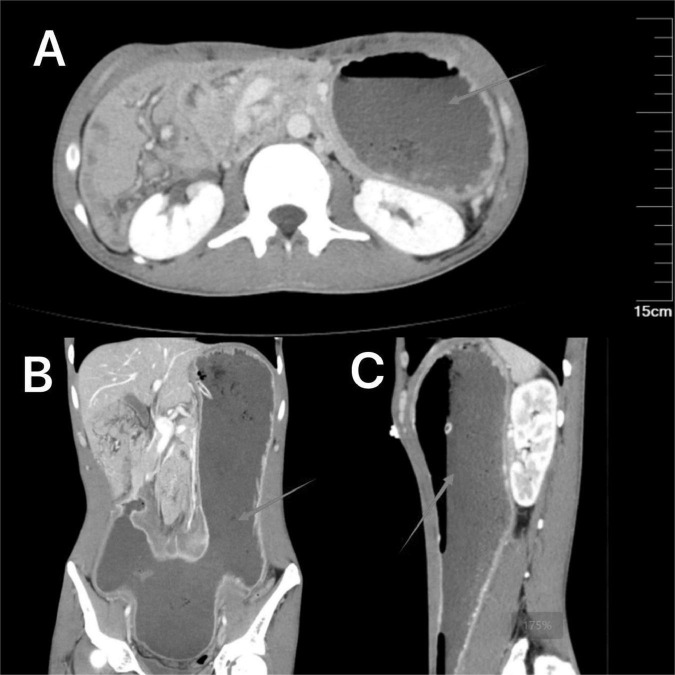
The enhanced CT scan images demonstrate the improvement in gastric dilation following the placement of a nasogastric tube. **(A)** (Axial view) shows a reduction in gastric dilation with the nasogastric tube in place. **(B)** (Coronal view) and **(C)** (sagittal view) further illustrate the improved gastric silhouette, confirming the effectiveness of the decompression.

Immediately after admission, a nasogastric tube was inserted to decompress the stomach, and approximately 2,000 mL of gastric fluid was drained. The patient’s symptoms and signs notably improved after fasting and nasogastric tube decompression. On hospital day 3, an upper gastrointestinal endoscopy was performed, revealing a pyloric ulcer with stenosis, gastric retention, and a relaxed cardia. During the patient’s hospitalization, a comprehensive treatment regimen was implemented, including anti-infection therapy, gastric mucosal protection, fluid resuscitation, parenteral nutritional support, fasting, and gastrointestinal decompression. These interventions led to a significant alleviation of the patient’s symptoms. A follow-up contrast-enhanced CT scan of the abdomen demonstrated improved gastric dilation, with marked stenosis at the pyloric region and no filling of the distal small intestine (Figure 2). On the fifth day of hospitalization, the patient requested discharge and was subsequently discharged in stable condition.

Throughout the hospitalization, the patient did not experience any adverse or unanticipated events. The interventions were well-tolerated, and there were no complications such as gastric perforation or necrosis noted during the hospital stay.

## Discussion

Acute gastric dilatation was first described by Duplay in 1833 (9). This clinical scenario can emerge subsequent to abdominal surgical procedures, physical trauma, or in the context of metabolic dysregulations and psychiatric disorders, such as diabetes mellitus and eating disturbances, including anorexia nervosa and bulimia nervosa. It is worth noting that there have been instances where the etiology of AGD could not be ascertained, which underscores the fact that the comprehensive pathophysiological mechanisms underlying AGD are still not fully elucidated (10, 11). While gastric expansion due to binge eating is well-documented, the precise mechanisms linking overeating to AGD remain incompletely understood. In this particular case, the patient had a history of chronic dyspepsia and had been taking TCM for a long time to regulate their condition. While the exact duration in this case was unspecified, the patient’s decade-long history of dyspepsia and self-reported “intermittent TCM use for symptom control” suggests cumulative exposure spanning multiple years. However, the excessive intake of TCM on this occasion turned out to be a triggering factor for acute gastric dilatation. This case should serve as a warning. It indicates that even TCM, which is intended to promote gastric motility and aid digestion, should not be taken blindly (12–15). Especially for patients with organic lesions or pyloric obstruction, caution must be exercised when using such medications. Furthermore, this highlights the need for a more comprehensive understanding of the interactions between different substances in TCM and their potential impacts on the gastrointestinal tract, as well as the importance of individualized treatment plans based on patients’ specific medical conditions to avoid such adverse outcomes.

Although the majority of reports on AGD associated with abnormal eating behaviors predominantly highlight excessive intake of solid food, While most reported AGD cases involve solid food binge-eating, our patient’s consumption of 3.5 liters of liquid TCM over 72 h (mean 1.166 liters/day) is particularly significant given his pre-existing conditions. For patients with chronic dyspepsia, pyloric obstruction, and chronic gastritis, such a high volume of liquid intake over a short period poses a significant risk of fluid overload and exacerbation of gastric symptoms (16, 17). While the temporal association between TCM consumption and AGD onset is evident, causality cannot be definitively established. Three potential mechanisms may explain this association: (1) volume overload: The total liquid intake (3.5 liters/72 h) likely exceeded gastric capacity, compounded by pyloric stenosis delaying emptying. (2) TCM composition: certain herbs (e.g., atropine-like alkaloids) may inhibit gastric motility, though the exact formulation was unrecalled. (3) Pre-existing conditions: chronic gastritis and pyloric stenosis created a “double-hit” effect, where fluid accumulation exacerbated anatomical obstruction. It is also important to consider whether excessive consumption of non-TCM substances, such as beverages or other fluids, could potentially lead to similar outcomes. While there are reports of AGD associated with binge eating and excessive intake of solid food, cases involving large volumes of liquid are less common (18). Diagnosing TCM-induced AGD is challenging. When considering the differential diagnosis, other potential causes of gastric dilatation, such as mechanical obstruction or systemic causes, should also be carefully evaluated. In this case, abdominal CT and endoscopy were instrumental in identifying pyloric obstruction as a contributing factor, helping to rule out other mechanical or systemic causes. For example, vomiting after taking large amounts of TCM may initially be mistaken for acute drug poisoning, leading doctors to overlook the diagnosis of gastric dilation. This can result in the omission of further diagnostic imaging, delaying essential decompression treatments and increasing the risk of severe complications such as gastric necrosis and perforation.

AGD has been mainly reported in younger patients, with few cases involving the elderly (19). Clinically, patients typically present with abdominal pain, vomiting, or bloating (20–23). However, there are cases, such as the one presented here, where the condition occurs without significant abdominal pain and bloating. The absence of pain, especially in younger individuals after taking large amounts of TCM, may lead clinicians to misinterpret symptoms as acute drug poisoning, potentially overlooking the more serious underlying condition of AGD.

The diagnosis of TCM-associated AGD requires a thorough exclusion of alternative causes. In this case, while pyloric stenosis and chronic gastritis were identified as contributing factors, other potential etiologies such as gastric volvulus or superior mesenteric artery syndrome were ruled out by the absence of specific CT findings like intestinal malrotation or vascular compression. Systemic conditions like diabetic gastroparesis seemed unlikely given the patient’s normal glucose tolerance and acute symptom onset. The temporal correlation between excessive TCM intake and symptom development, along with the exclusion of these competing diagnoses, strengthens the plausibility of TCM-induced gastroparesis in the context of pre-existing anatomical vulnerability. Notably, the patient’s history of intermittent TCM use without prior complications suggests that the sheer volume of ingestion (3.5 liters over 72 h) rather than specific herbal toxicity may have been the primary trigger.

## Conclusion

This report documents a unique case of acute gastric dilatation (AGD) in a young male patient with chronic dyspepsia requiring prolonged intermittent herbal therapy. Crucially, the patient exhibited no history of abdominal surgery, diabetes mellitus, or eating disorders. The AGD episode manifested as an acute abdominal emergency without preceding mechanical obstruction. Of particular clinical significance, the temporal correlation between symptom onset and excessive ingestion of herbal decoctions—coupled with the exclusion of conventional etiologies—suggests potential phytochemical-induced gastroparesis as the underlying mechanism. This contrasts with typical AGD cases associated with solid food impaction or binge eating behaviors, thereby expanding the recognized spectrum of medication-related gastrointestinal dysmotility disorders.

This case highlights the need for a more comprehensive understanding of the interactions between different substances in TCM and their impact on the gastrointestinal tract. Additionally, it suggests that excessive fluid intake, regardless of the type of fluid, can lead to AGD in susceptible individuals. However, a limitation of this report is the lack of specific details about the TCM formulation ingested, as the patient could not recall the herbal ingredients. Future cases should document TCM components to assess their pharmacological effects on gastrointestinal motility. Clinicians may attribute symptoms solely to acute drug poisoning and overlook the need for abdominal imaging or nasogastric tube placement. Such oversight can delay the diagnosis of AGD, increasing the risk of severe complications like gastric wall necrosis and perforation.

This case illustrates how acute gastric dilatation (AGD) developed through the interplay of three key factors: the patient’s excessive consumption of traditional Chinese medicine (approximately 3.5 liters over 72 h), pre-existing pyloric stenosis, and chronic gastritis. While the temporal relationship with TCM intake is clinically significant, it’s important to recognize that similar outcomes could potentially occur with other types of fluid overload in anatomically vulnerable individuals. This observation underscores the necessity of comprehensive clinical evaluation that considers both structural abnormalities and functional impairments in patients presenting with acute gastric symptoms.

Moving forward, this case highlights several important research needs in understanding herbal medicine-related complications. Priority should be given to pharmacological studies examining the effects of common TCM components, particularly those with known anticholinergic properties, on gastrointestinal motility. Clinical research should employ objective measures like gastric scintigraphy to track motility changes in chronic TCM users with pre-existing digestive disorders. The establishment of an international case registry could significantly advance our understanding by systematically documenting key variables such as dosage patterns, preparation methods, and concomitant medications. These research efforts would benefit from collaborative approaches that combine Western medical expertise with traditional medicine knowledge. In clinical practice, physicians should maintain detailed records of herbal formulations, including their composition and preparation methods, when evaluating similar cases of unexplained gastric distress.

## Data Availability

The raw data supporting the conclusions of this article will be made available by the authors, without undue reservation.
